# Supplementary material for: Causal Relationship between Aging and Anorexia Nervosa: A White-Matter-Microstructure-Mediated Mendelian Randomization Analysis
Source: Biomedicines. 2024 Aug 16;12(8):1874. doi: 10.3390/biomedicines12081874 (PMC11351342; doi:10.3390/biomedicines12081874)
Supplement: Supplementary file 1 [file biomedicines-12-01874-s001.zip › Supplementary Figure/Figure S4/Singe SNP plot of MR analysis for TL to ubm-b-2113.pdf]

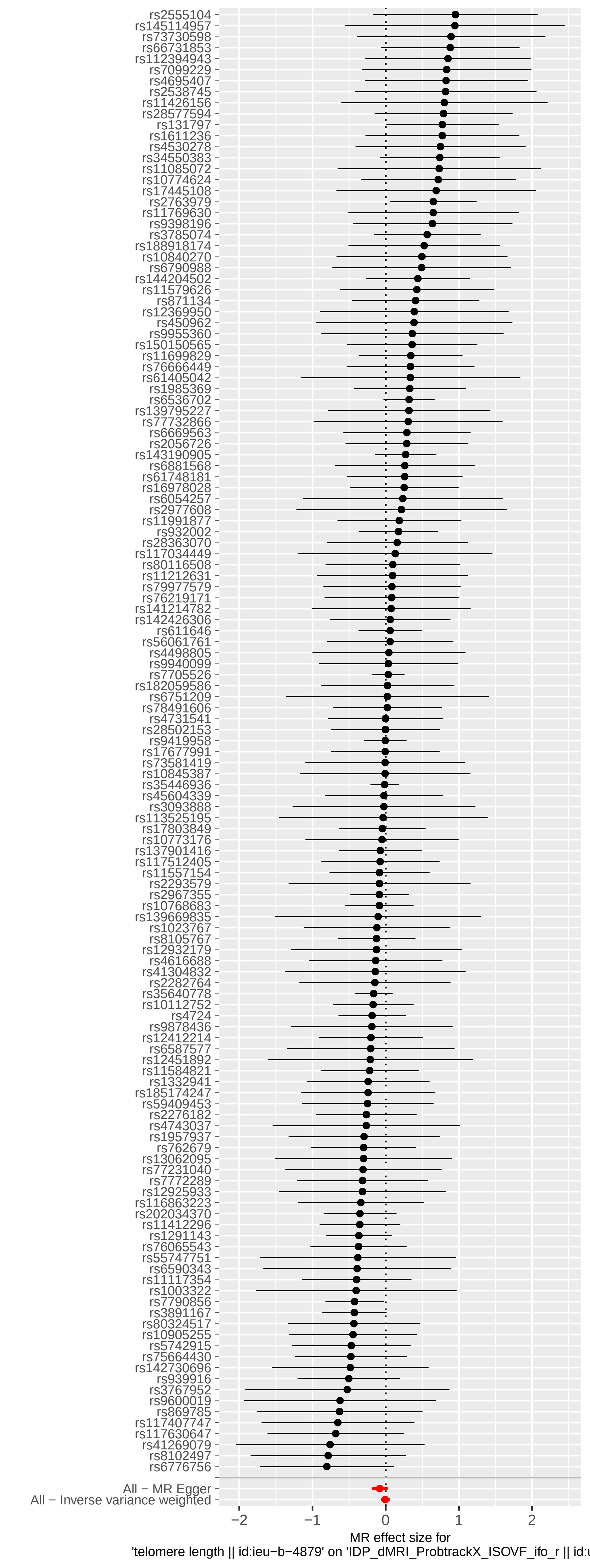
rs2555104
rs145114957
rs73730598
rs66731853
rs112394943
rs7099229
rs4695407
rs2538745
rs11426156
rs28577594
rs131797
rs1611236
rs4530278
rs34550383
rs11085072
rs10774624
rs17445108
rs2763979
rs11769630
rs9398196
rs3785074
rs188918174
rs10840270
rs6790988
rs144204502
rs11579626
rs871134
rs12369950
rs450962
rs9955360
rs150150565
rs11699829
rs76666449
rs61405042
rs1985369
rs6536702
rs139795227
rs77732866
rs6669563
rs2056726
rs143190905
rs6881568
rs61748181
rs16978028
rs6054257
rs2977608
rs11991877
rs932002
rs28363070
rs117034449
rs80116508
rs11212631
rs79977579
rs76219171
rs141214782
rs142426306
rs611646
rs56061761
rs4498805
rs9940099
rs7705526
rs182059586
rs6751209
rs78491606
rs4731541
rs28502153
rs9419958
rs17677991
rs73581419
rs10845387
rs35446936
rs45604339
rs3093888
rs113525195
rs17803849
rs10773176
rs137901416
rs117512405
rs11557154
rs2293579
rs2967355
rs10768683
rs139669835
rs1023767
rs8105767
rs12932179
rs4616688
rs41304832
rs2282764
rs35640778
rs10112752
rs4724
rs9878436
rs12412214
rs6587577
rs12451892
rs11584821
rs1332941
rs185174247
rs59409453
rs2276182
rs4743037
rs1957937
rs762679
rs13062095
rs77231040
rs7772289
rs12925933
rs116863223
rs202034370
rs11412296
rs1291143
rs76065543
rs55747751
rs6590343
rs11117354
rs1003322
rs7790856
rs3891167
rs80324517
rs10905255
rs5742915
rs75664430
rs142730696
rs939916
rs3767952
rs9600019
rs869785
rs117407747
rs117630647
rs41269079
rs8102497
rs6776756
All − MR Egger
All − Inverse variance weighted
−2
−1
0
1
2
MR effect size for
'telomere length || id:ieu−b−4879' on 'IDP_dMRI_ProbtrackX_ISOVF_ifo_r || id:u
